# Formation of Nanospikes on AISI 420 Martensitic Stainless Steel under Gallium Ion Bombardment

**DOI:** 10.3390/nano9101492

**Published:** 2019-10-19

**Authors:** Zoran Cenev, Malte Bartenwerfer, Waldemar Klauser, Ville Jokinen, Sergej Fatikow, Quan Zhou

**Affiliations:** 1 Department of Electrical Engineering and Automation, School of Electrical Engineering, Aalto University, Maarantie 8, 02150 Espoo, Finland; zoran.cenev@aalto.fi; 2 Department of Computing Science, University of Oldenburg, Ammerländer Heerstraße 114-118, 26129 Oldenburg, Germany; waldemar.klauser@uni-oldenburg.de (W.K.); sergej.fatikow@uni-oldenburg.de (S.F.); 3 Department of Chemistry and Materials Science, Aalto University, School of Chemical Engineering, Tietotie 3, 02150 Espoo, Finland

**Keywords:** focused ion beam, nanospikes, martensite, stainless steel, gallium, bombardment, irradiation effects, sharp needle, incident angle

## Abstract

The focused ion beam (FIB) has proven to be an extremely powerful tool for the nanometer-scale machining and patterning of nanostructures. In this work, we experimentally study the behavior of AISI 420 martensitic stainless steel when bombarded by Ga^+^ ions in a FIB system. The results show the formation of nanometer sized spiky structures. Utilizing the nanospiking effect, we fabricated a single-tip needle with a measured 15.15 nanometer curvature radius and a microneedle with a nanometer sized spiky surface. The nanospikes can be made straight or angled, depending on the incident angle between the sample and the beam. We also show that the nanospiking effect is present in ferritic AISI 430 stainless steel. The weak occurrence of the nanospiking effect in between nano-rough regions (nano-cliffs) was also witnessed for austenitic AISI 316 and martensitic AISI 431 stainless steel samples.

## 1. Introduction

The focused ion beam (FIB) technique has been established as a powerful tool for micro and nanoscale imaging [1], sputtering, deposition [2], 3D machining [3], and surface modifications [4]. When an incident ion comes into contact with a targeted material, the ion enters into a set of collisions (higher than normal thermal energies) with the target atoms, a process known as a collision cascade. Sputtering occurs when an incident ion comes into contact with a targeted surface and transfers its momentum to the host atoms. A host atom on the surface will absorb a part of the ion’s kinetic energy. If the new energy state of the host surface atom is higher than the surface binding energy (SBE) of the targeted material, then the surface atom will be ejected as a sputtered particle [5]. A quantitative measure of sputtering is defined through sputtering yield, i.e., the number of atoms removed by an incident ion. The sputtering yield is affected by the material composition, angle of incidence, the crystal structure of the substrate, redeposition, scanning speed, temperature of the target, and surface contaminations [6].

The process which constrains the path of the ion in a crystalline solid is known as ion channeling [7]. Along low index directions in crystalline materials, ions may penetrate greater distances as compared to cascade collisions in amorphous materials. Since ion channeling has a direct impact on the ion penetration range, meaning the trajectory within the collision cascade, it also impacts the sputtering yield. Variations on sputtering yield within a sample target cause roughening of the surface, which has been observed for aluminum [8], tungsten [9], and polycrystalline gold [10]. A nanometer sized spiky structure, an extreme form of nano-roughening, with distinct and visibly pronounced spikes, occurs during the anisotropic etching of single crystal (100) copper [8], tungsten [11], and 18 Cr-ODS (Oxide Dispersive Strengthened) steel [12]. Pyramidal and conical (faceted pyramid) micro/nanometer-sized structures have been observed much earlier on tin crystals [13], and monocrystalline [14] and polycrystalline copper [15] when irradiated by argon, as well as krypton ions [16]. The origins and stability of ion-bombarded copper surfaces have been heavily analyzed and discussed by Auciello and Kelly [17,18].

Here, we experimentally demonstrate the formation of nanospikes occurring on a martensitic AISI 420 stainless steel surface when bombarded with gallium ions. We also show that nanospikes can be made straight or angled depending on the incident angle of the FIB. To demonstrate potential applications, we FIB-treated an electrochemically etched stainless steel (AISI 420) tip to induce nanospiking and thus obtain a single tip nano-needle with a measured diameter of 15.15 nanometers. Additionally, we FIB-treated an electrochemically etched stainless steel (AISI 420) tip with micrometer sharpness to induce nanospikes. Finally, we also show that the nanospiking effect is present in ferritic AISI 430 stainless steel. The weak occurrence of the nanospiking effect in between nano-rough regions (nano-cliffs) was also witnessed for austenitic AISI 316 and martensitic AISI 431 stainless steel samples.

Future research should focus on using the single sharp nano-needle for creating localized magnetic fields, as in [19], or laser-induced electron emission, as in [20,21]. Due to the soft magnetic properties of the martensitic ASI 420 stainless steel, nanometer sized spiky magnetic tips could be applied in producing magnetic nano-devices, for example, magneto-gravitational traps [22,23]. Another line of research can focus on producing superhydrophobic/hydrophobic microneedles by subsequent fluoropolymer deposition (low adhesive polymer) to the surface of the microneedle with the nanometer sized spiky surface.

## 2. Materials and Methods

### 2.1. Procedure of FIB Treatment of Martensitic, Austenitic and Ferritic Stainless Steel Plates

The treatment of the martensitic AISI 420 (Fe-86,7/Cr13,0/C0,3) stainless steel sample (Goodfellow, Cambridge, UK) was carried out with a dual-beam high-resolution scanning electron/focused ion beam microscope, namely, the Lyra FEG (TESCAN, Brno, Czech Republic). A 0.5 cm^2^ piece was cut from the foil sheets. The piece was cleaned in an ultrasonic isopropanol bath, with a 10 min O_2_ plasma treatment under 40 kHz at 100 W in the plasma system, using the Femto instrument (Diener electronic GmbH and Co KG, Ebhausen, Germany). An area of 10 µm^2^ was exposed to an ion dose of 19.4 nC/µm^2^ at a 30 keV beam energy and emission current of 2 µA. The same treatment was applied for the other stainless steel samples, i.e., AISI316 (Fe/Cr18/Ni10/Mo3), AISI430 (Fe81/Cr17/Mn/Si/C/S/P) and AISI431 (Fe82/Cr16/Ni2), as were received from the supplier (Goodfellow, UK).

### 2.2. Procedure of Fabrication of Martensitic Stainless Steel Needle with Nanometer Sharpness

A one millimeter thick stainless steel AISI 420 wire (Goodfellow, Cambridge, UK) was thinned with up to micrometer sharpness, as previously reported in [19]. The etched needle was installed into the FIB-SEM dual beam system (Lyra FEG) and is shown in Figure S1a–c. Prior to FIB exposure, the needle was cleaned in an ultrasonic acetone bath and rinsed with isopropanol. A series of FIB exposures with a total ion dose of roughly equal to 2000 nC/µm^2^ at a 30 keV beam energy and emission current of 2 µA was applied in order to induce more spikes (Figure S1d). Once a prominent spike was obtained, it was isolated from further exposure, but the exposure was targeted towards removing the surrounding spikes and eventually providing the final result (Figure S1f).

### 2.3. Procedure of Fabrication of Stainless Steel Microneedle with Nanospikes

A one millimeter thick stainless steel AISI 420 wire (Goodfellow, USA) was installed into the collet of a milling 3-axis bridge router, as illustrated in Figure S2a. A face mill insert with four cutting edges was used for machining, where the wire would be thinned within a range of 0.4 and 0.7 mm thickness, with a length of about 3 mm. A thinned wire as such was mounted onto a holder of an in-house built electrochemical etching station, containing a 10% HCl bath, a computer-controlled voltage supply, and a motorized stage (further details can be found in [19]). The first step was electrochemical thinning, consisting of dipping the wire 3 mm into the HCl bath. The etching started when a voltage of 1V was supplied. Immediately after voltage application, the wire was pulled with a constant speed of 10 µm/s. The second etching step consisted of re-dipping the wire by 1 mm, with supply voltage of 1V and pulling the wire with a constant speed of 10 µm/s until the needle was completely out of the bath, as illustrated in Figure S2b. A sample micrograph of an etched needle can be seen in Figure S3a–c.

The electrochemically etched needle was installed into the FIB-SEM dual beam system, as illustrated in Figure S2c. Prior to FIB treatment, the machined/etched needles were cleaned in an ultrasonic acetone bath and rinsed with isopropanol. The needles were exposed to an ion dose of 10.6 nC/µm^2^ at a 30 keV beam energy and emission current of 2 µA (Figure S3d,e).

## 3. Results

### 3.1. Nanospikes Formation on AISI 420 Martensitic Stainless Steels by FIB Treatment

**Figure 1:**
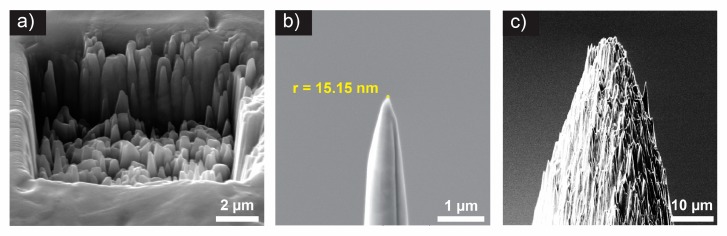
Nanospiking effects on martensitic AISI 420 stainless steel. (**a**) Nanometer sized spiky surface of the martensitic AISI 420 stainless steel sample plate after FIB treatment with gallium ions with a dose of 19.4 nC/µm^2^. Fabrication results of a (**b**) sharp needle with nanometer resolution, the circle denotes fitting to the curvature of the tip. The original raw image without fitting is given in Figure S1f. (**c**) A micrometer-scale needle with nanospikes.

Figure 1a shows the surface morphology of an FIB-irradiated AISI 420 sample with gallium ions. Details of the sample preparation and FIB treatment settings are provided in Section 2.1. From the figure, it can be seen that the sharpness of the nanospikes is in the sub-micron range. One can also see that the nanospikes on the edge feature higher aspect ratios than the nanospikes in the middle of the trench.

To demonstrate the potential usability of the nanospiking effect, we have fabricated two different types of needles, i.e., an extremely sharp needle with radius of 15.15 nanometers (Figure 1b) and a micrometer-sized needle, featuring a nanometer sized spiky topology (Figure 1c). The fabrication procedure of both needles is similar, and they are explained in detail in Section 2.2 and Section 2.3, respectively. One should note that the fabrication procedure for both needles includes a certain level of randomness, however, the sharpness of the nanospikes is very often in the low nanometer range (from several up to tens of nanometers).

### 3.2. Morphological Evolution of AISI 420 during FIB Treatment

**Figure 2:**
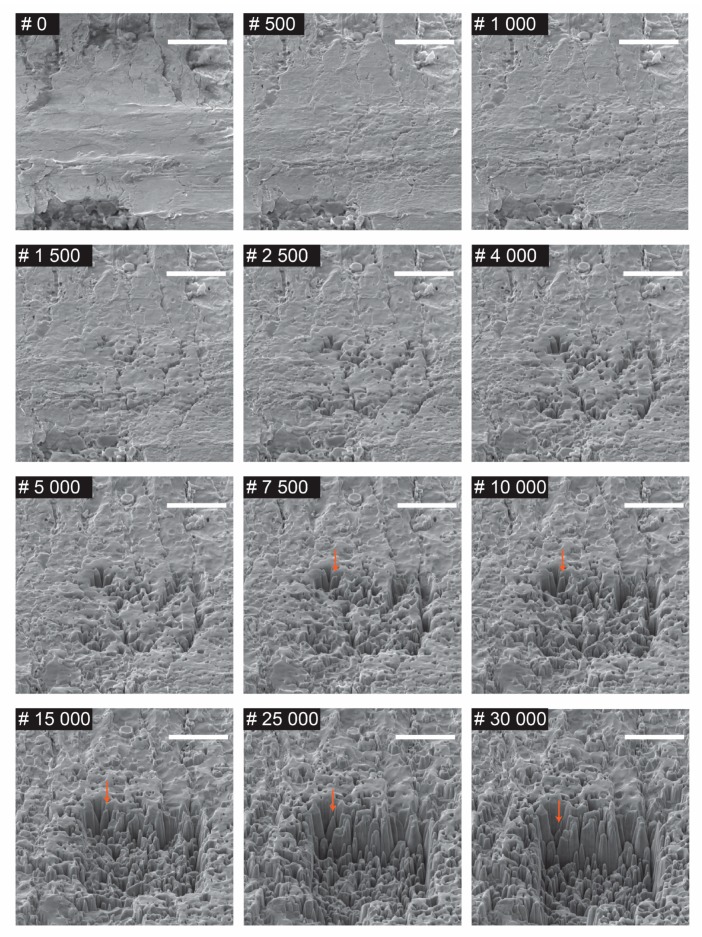
Evolution of nanospikes at normal incidence as a function of FIB dose from 0 to 30,000 scans (1000 scans correspond to a gallium ion dose of 1435 nC/µm^2^). The spiky structure shift downwards along with increase of the FIB exposure. Scale bar in each image is 5 µm. Orange arrows denote the formation and size variation of the firstly formed nanospike.

We also examined the morphological evolution of the martensitic AISI 420 in a step-by-step manner. Figure 2 shows the surface morphology evolution, and finally, the formation of the nanospikes. The samples were exposed to 30,000 scans overall (1000 scans correspond to an ion dose of 1435 nC/µm^2^). The trench dimension was 10 × 10 µm^2^. The AISI 420 surface was untreated at the beginning (0 scans), and after the first 500 scans, the appearance of a few pits on the surface was noted.

The indentation of the initial pits increased with the increase of the number of scans (1000 to 5000 scans). At 7500 scans, the formation of the first nanospike (denoted with an orange arrow) was noticed. Further exposure of the earlier formed nanospikes causes their increase in sharpness, but also causes a decrease in height, as indicated by the orange arrows (10,000 to 30,000 scans). With further increase of the irradiation, new spikes started to form, and they could be found more within the central region of the trench, rather than on the edges. The nanospikes on the edges feature much higher aspect ratios than the ones in the central region. This difference in aspect ratios can be observed from scans 15,000 to 30,000.

### 3.3. Energy-Dispersive X-ray Spectroscopy (EDX) and X-ray Photoelectron Spectroscopy (XPS) Analysis of FIB Irradiated AISI 420 Stainless Steel Alloy with Gallium Ions

We have performed energy-dispersive X-ray spectroscopy (EDX) analysis on the whole gallium irradiated trench, a spot on a single nanospike, and a non-irradiated area (Figure S4). The only difference that the EDX results show is the presence of gallium in irradiated regions in comparison to non-irradiated regions. No significant change in the presence of the iron or chromium content within the AISI 420 sample before and after gallium irradiation was determined.

We also have performed X-ray photoelectron spectroscopy (XPS) measurements with the Kratos Axis Ultra ESCA system (Kratos Analytical Ltd., Manchester, UK), analyzing the gallium irradiated circular trench (diameter of ~35 µm) and the non-irradiated area (Figure S5). The XPS results show a reduction of iron (9.42% to 2.2% for XPS aperture of 27 µm) and chromium (0.97% to 0.44% for XPS aperture of 27 µm) between the non-irradiated and irradiated regions. Here, it could be that the gallium in the non-irradiated region was deposited during the gallium irradiation of the sample in the FIB system. The existence of high oxygen and carbon concentrations is due to exposure of the sample to ambient conditions.

### 3.4. Effect from Variation of Incident Angle

**Figure 3:**
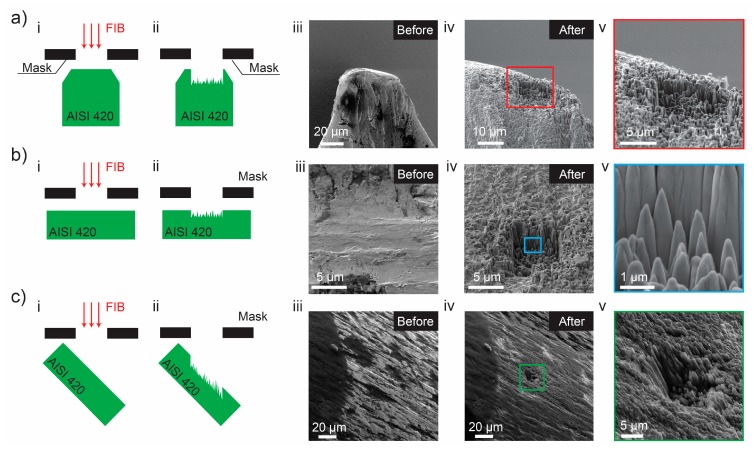
Spiking phenomena of AISI 420 stainless steel sample probes during gallium irradiation with different incident angles: (**a**) Vertical position of the probe; (**b**) Horizontal position of the probe; and (**c**) at an incident angle of 40° to the probe. Here, (**i**) and (**ii**) illustratively depict the orientation of the sample, the gallium irradiated regions and the spiking result, respectively. Here, (**iii**) and (**iv**) show the results obtained before and after the FIB irradiation. Here, (**v**) are close-ins of (**iv**).

The effect from the variation of the incident angle has been studied by the different orientation of an AISI 420 stainless steel probes during gallium irradiation (Figure 3). At first, a probe was installed in a vertical position (Figure 3a) and it was subjected to gallium irradiation in FIB system. After a pre-defined FIB dose was delivered to the probe, the nanospikes formed in the direction of the beam. The same nanospikes formation occurred for a horizontally positioned probe (Figure 3b) and 40° inclined probe (Figure 3c). From these results, one can infer that nanospikes form regardless of the incident angle in this specific martensitic steel alloy.

### 3.5. Nanospiking Effect on Austenitic AISI 316, Ferritic AISI 430 and Martensitic AISI 431 during FIB Treatment

Figure S6 shows a comparison of FIB irradiated stainless steel plates with gallium ions of other three different stainless steel types, i.e., austenitic (AISI 316), ferritic (AISI 430) and martensitic (AISI 431) stainless steel plates. Details of the sample preparation and FIB treatment are provided in Section 2.1. All samples have an anisotropic etching behavior. The austenitic AISI 316 stainless steel sample (a and d) shows a mix of inhomogeneous nano-rough regions and regions with nanospikes (Figure S6d). Nano-rough regions look like mountain range or cliffs, therefore the notation “nano-cliffs”, for instance, see the orange arrows in Figure S6. The ferritic AISI 431 (c and e) features only a region with nanospikes (Figure S6e). The AISI 431 displays a presence of nanospikes, however, these are seldom scattered in between the nano-roughed bottom (Figure S6f).

## 4. Discussion

As can be seen from Figure 2 and Figure 3, the nanospikes on the edges have much higher aspect ratios than the ones in the central region of the trench. When sputtering occurs at the edges, it nucleates the edges (formation of nanospikes), due to the presence of the gallium ions outside of the beam spot (the beam power features Gauss distribution). The inner part of the trench continues to be sputtered, but the part outside of the trench is slightly affected by the satellite gallium ions. The satellite gallium ions also cause sputtering, but at significantly reduced rates than the inner part of the trench. The sputtering continuity of the inner part shifts the nano-spiky structure downwards into the bulk, but this shift barely occurs at the edges. This discrepancy in the structural shift can explain why the nanospikes in the edges feature higher aspect ratios than the nanospikes in the central region of the trench.

Polycrystalline alloys such as the martensitic AISI 420 stainless steel (and the other FIB treated stainless steels) investigated in this work, besides the difference in material content, feature domains with different crystallographic orientations. The sputtering rates of neighboring domains may vary greatly, depending on the structural configuration of the grains and the orientation of the lattices in the particular domain with respect to the incident ion beam. However, we have shown that nanospikes occurred in the gallium bombarded AISI 420 sample, but not as much in the AISI 431 sample, where nano-cliffs were more dominant, although both samples are martensitic stainless steels with very similar crystalline structure [24,25].

We have performed EDX and XPS analysis to investigate the material content within the non-irradiated and the irradiated regions of the martensitic AISI 420 stainless steel alloy. The EDX results (penetration depth up to 10 µm) show the presence of iron, chromium, and gallium in the gallium-irradiated regions. The XPS results show a decrease of the iron and the chromium in the irradiated trench with respect to non-irradiated surface. The XPS results do not indicate any saturation of a single element on the very surface in the irradiated regions.

Other studies have demonstrated that ion channeling affects the sputtering yield in polycrystalline materials such as [4,8,9], therefore inducing nano-roughening on the treated surface. However, we are not sure whether the same explanation can be attributed to the formation of the nanospikes. The nanometer sized spiky formations are special forms of the nano-roughed surface, and the exact mechanism has been recently discussed by Prenitzer et al. [8] and Ran et al. [12], but also heavily researched much earlier [13,14,15,16,17,18]. Auciello [18] claims that the micro/nanometer scaled pyramidal structures form due to sputtering differences in (1) the presence of intrinsic and/or bombardment-induced sub-surface defects, (2) the evolution of pre-existing and/or bombardment-induced asperities of convex-up curvatures, and (3) the erosion of nuclei formed by migration of sputter-deposited foreign atoms on the substrate of the surface. The observed nanospike formations by Prenitzer et al. [8] were attributed to a wide range of sputtering conditions, whereas the most likely one may be the quality of initial target surface. Ran et al. [12] imaged 18 Cr-ODS steel nanospikes with transmission electron microscopy (TEM), showing that two different crystal orientations do exist in one nanospike with distinct two grains and a clear grain boundary. The report claims that nanospike formation is not induced by grain recrystallization and regrowth during Ga^+^ ion bombardment, but rather due to an interplay between a curvature-dependent sputtering and defect accumulation near the surface. Both reports address the importance of the initial surface topology. This interplay between a curvature-dependent sputtering and defect accumulation near the surface seems to be a valid argument and might be used to interpret our experimental observations, since the morphological variation of the targeted surfaces greatly impacts the dynamic competition of available atoms on the substrate, the atom evacuation due to sputtering, and the gathering of vacancies.

## 5. Summary and Conclusions

The nanospiking phenomenon has been previously reported for copper [8], tungsten [11], and 18Cr-ODS steel [12]. In this communication, we have shown that nanospikes are formed on martensitic AISI 420 stainless steel when treated with FIB. The nanospikes can be made straight or angled depending on the incident angle between the sample and the beam. We also showed fabrication of a <16 nanometer sharp single tip needle and a micrometer-sized sharp needle with nanospikes. The nanospiking effect occurs in ferritic AISI 430 stainless steel sample too. A weak occurrence of the nanospiking effect in between nano-rough regions (nano-cliffs) was also witnessed for the austenitic AISI 316 and martensitic AISI 431 stainless steel samples. Unlike the intermediate existence of the nano-pyramidal structures reported in [16], the nano-spiky structures reported here are stable and occur at different irradiation doses.

The nanospiking phenomenon in martensitic AISI 420 stainless steels has promising capacity for future research. The single sharp nano-needle has potential of being used for creating localizing magnetic fields, as in [19], or laser-induced electron emission as in [20,21]. A micrometer-scaled needle with nano-spiky topology could be utilized for making superhydrophobic needles, performing droplet manipulation on open hydrophobic and superhydrophobic surfaces, where needle-droplet adhesion is less than droplet-substrate adhesion, similar to as in [26,27]. Since the martensitic stainless steel has soft ferromagnetic properties, the Ga^+^ ion bombardment process can be used for fabricating magnetic nanospikes, which might find application in the development of novel quantum devices, e.g., magneto-gravitational traps [22,23].

## Supplementary Materials

The following are available online at https://www.mdpi.com/2079-4991/9/10/1492/s1, Figure S1: Intermediate steps of the fabrication process of martensitic stainless steel AISI420 needle with nanometer sharpness. Figure S2: Illustration of the fabrication procedure of microneedle with nanospikes. Figure S3: Intermediate steps of the fabrication process of martensitic stainless steel AISI420 microneedle with nanospikes. Figure S4: Energy-dispersive X-ray spectroscopy (EDX) results of (a) the completely irradiated trench; (b) a spot on a single nanospike; (c) non-irradiated area. Figure S5: X-ray Photoelectron Spectroscopy (XPS) results of (a) a ~35 µm in diameter gallium irradiated trench; (b) non-irradiated area. i and ii denote measurement with XPS aperture of 27 and 55 µm, respectively. Figure S6: Gallium irradiation of austenitic AISI 316 (a,d), ferritic AISI 430 (b,e), and martensitic AISI 431 (c,f) stainless steel plates with a dose of 19.4 C/µm^2^. (a–c) Before and (d–f) after gallium irradiation.
