# Supplementary material for: Formation of Nanospikes on AISI 420 Martensitic Stainless Steel under Gallium Ion Bombardment
Source: Nanomaterials (Basel). 2019 Oct 19;9(10):1492. doi: 10.3390/nano9101492 (PMC6836003; doi:10.3390/nano9101492)
Supplement: Supplementary file 1 [file nanomaterials-09-01492-s001.pdf]

nanomaterials

MDPI

Supplementary Materials

Formation of Nanospikes on AISI 420 Martensitic Stainless Steel under Gallium Ion Bombardment

Zoran Cenev [1], Malte Bartenwerfer [2,*], Waldemar Klauser [2], Ville Jokinen [3], Sergej Fatikow [2] and Quan Zhou [1,*]

[1] Department of Electrical Engineering and Automation, School of Electrical Engineering, Aalto University, Maarantie 8, 02150 Espoo, Finland; zoran.cenev@aalto.fi
[2] Department of Computing Science, University of Oldenburg, Ammerländer Heerstraße 114-118, 26129 Oldenburg, Germany; waldemar.klauser@uni-oldenburg.de (W.K.); sergej.fatikow@uni-oldenburg.de (S.F.)
[3] Department of Chemistry and Materials Science, Aalto University, School of Chemical Technology, Tietotie 3, 02150 Espoo, Finland; ville.jokinen@aalto.fi
* Correspondence: m.bartenwerfer@uni-oldenburg.de (M.B.); quan.zhou@aalto.fi (Q.Z.); Tel.:+49-179-682-1971 (M.B.); +358-40-855-0311 (Q.Z.)

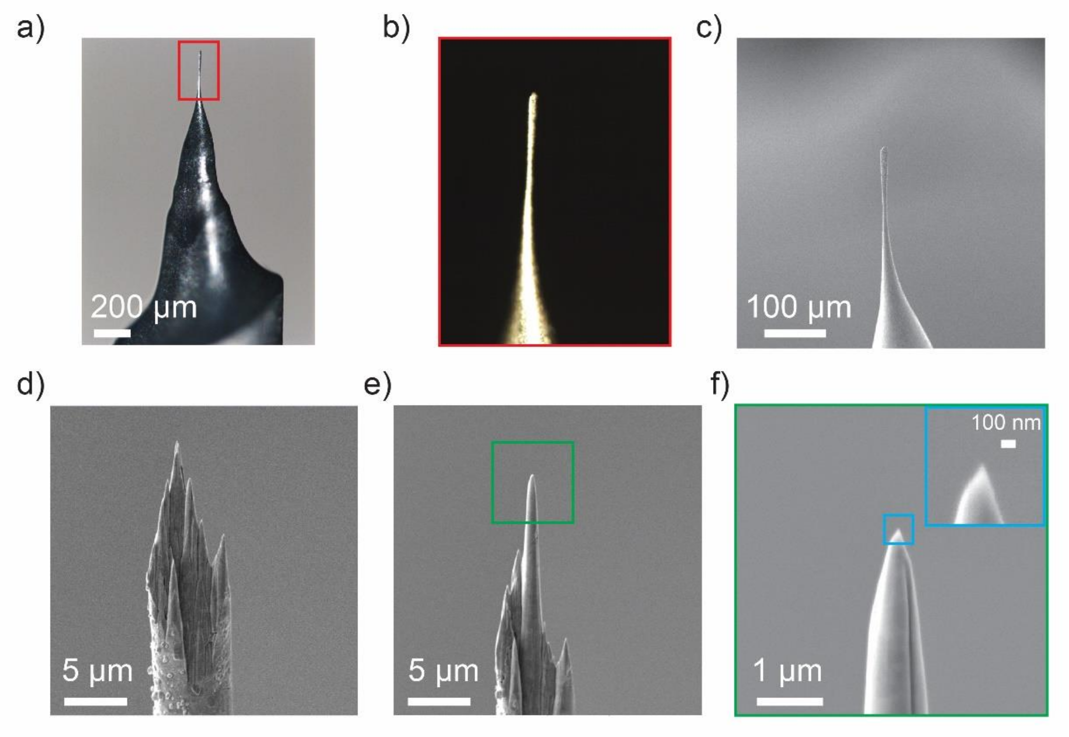

Figure S1. Intermediate steps of the fabrication process of martensitic stainless steel AISI420 needle with nanometer sharpness. a) Electrochemically thinned AISI 420 wire; b) close-up; c) SEM micrograph of the micrometer sharp tip before gallium irradiation. d) Nano-spikes formed after gallium irradiation in FIB/SEM dual system; e) FIB (with gallium ions) machining of surrounding spikes while isolating the middle one; f) The final nanospike with measured 15.15 nm diameter. Fitted circle indicating the measurement is given in Figure 1b, the inset is showing a close-in.

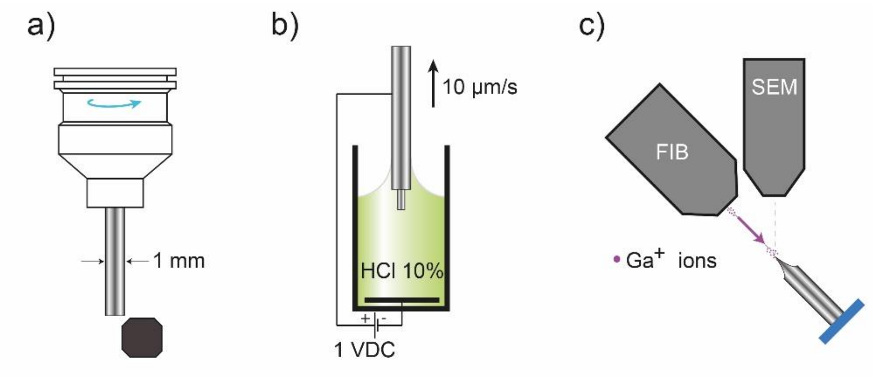

Figure S2. Illustration of the fabrication procedure of microneedle with nanospikes. a) Thinning by machining; b) Electrochemical etching in HCl bath with a constant speed of 10 μm/s; c) Gallium irradiation within FIB/SEM dual system.

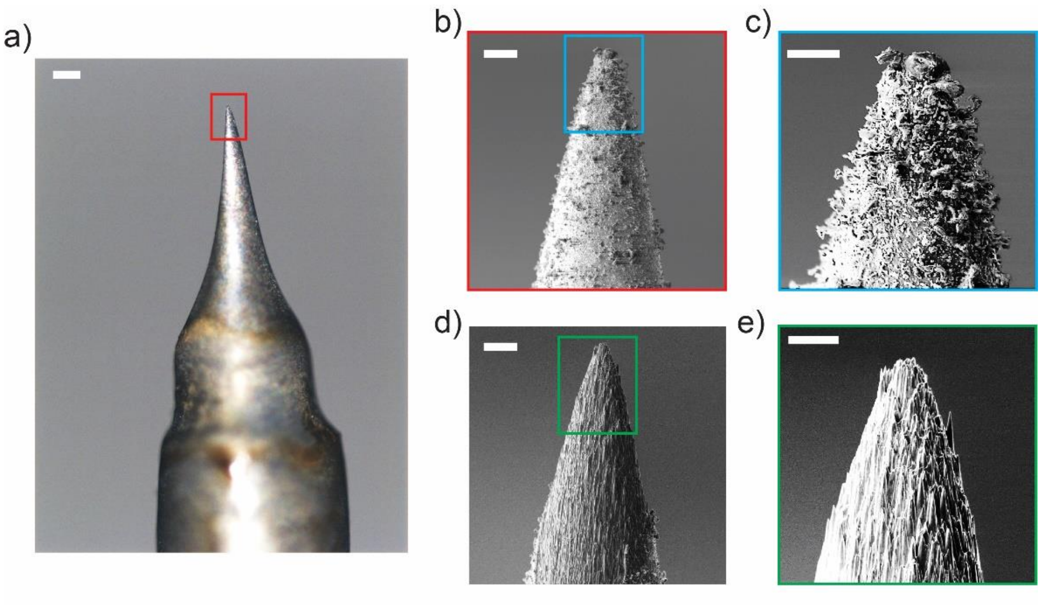

Figure S3. Intermediate steps of the fabrication process of martensitic stainless steel AISI420 microneedle with nanospikes. a) Thinned AISI 420 wire by machining and electrochemical etching; b) SEM micrograph of the micrometer sharp tip; c) close-up of b). d) SEM micrograph of the gallium irradiated tip. e) close-up of d). Scale bar is 100 μm in a), 20 μm in b) and d) and 10 μm in c) and e).

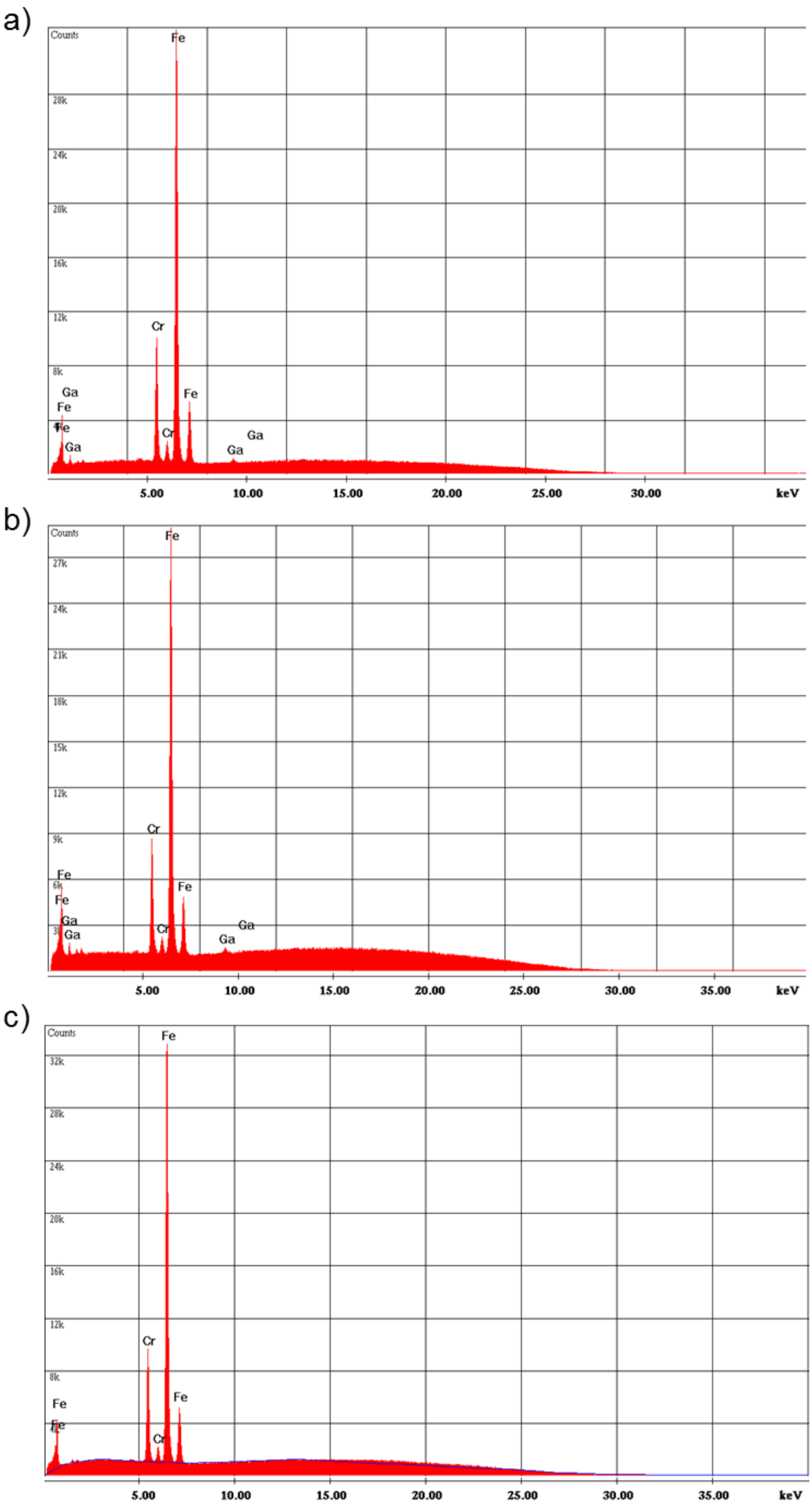

Figure S4. Energy-dispersive X-ray spectroscopy (EDX) results of a) the completely irradiated trench; b) a spot on a single nanospike; c) non-irradiated area.

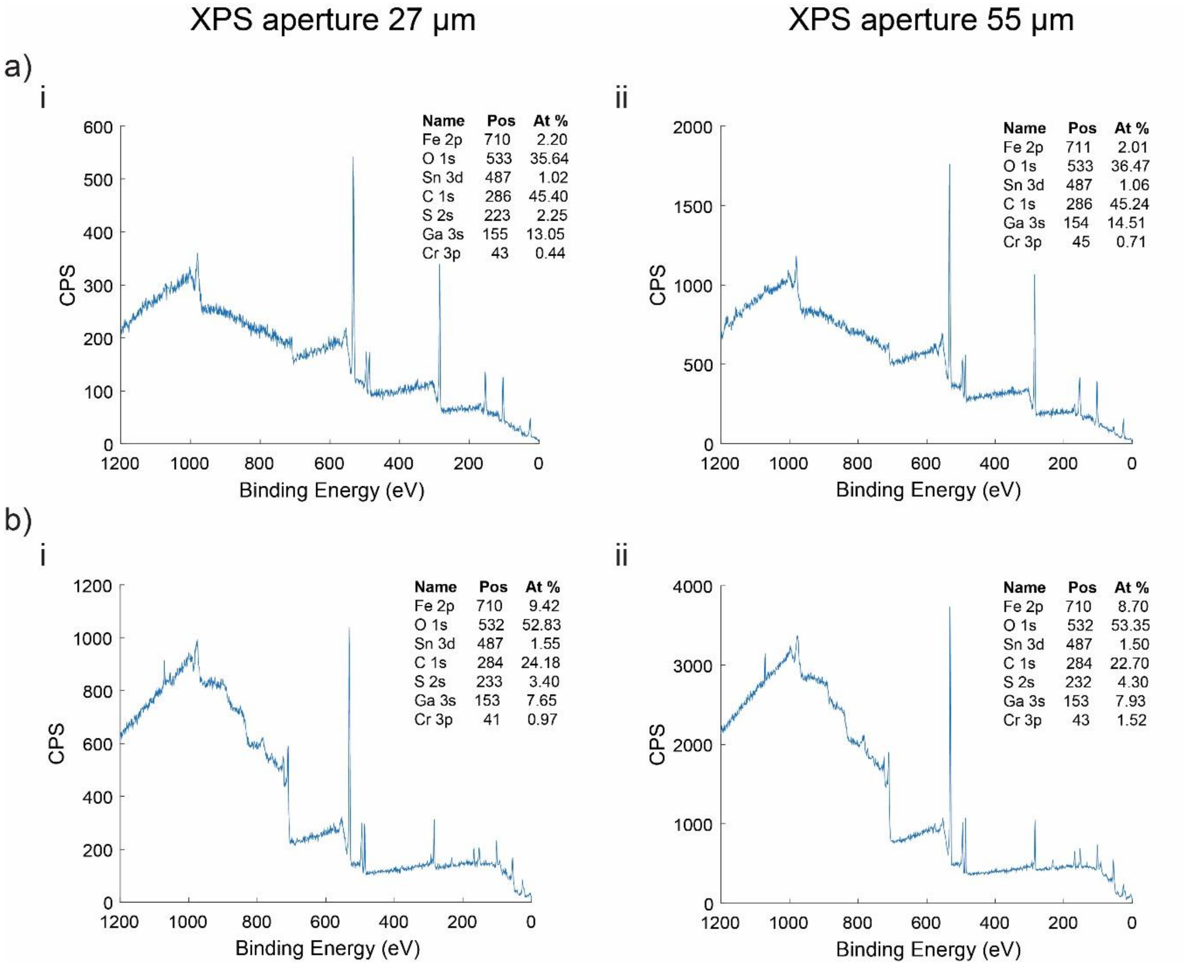

Figure S5. X-ray Photoelectron Spectroscopy (XPS) results of a) a ~35 µm in diameter gallium irradiated trench; b) non-irradiated area. i and ii denote measurement with XPS aperture of 27 and 55 µm, respectively.

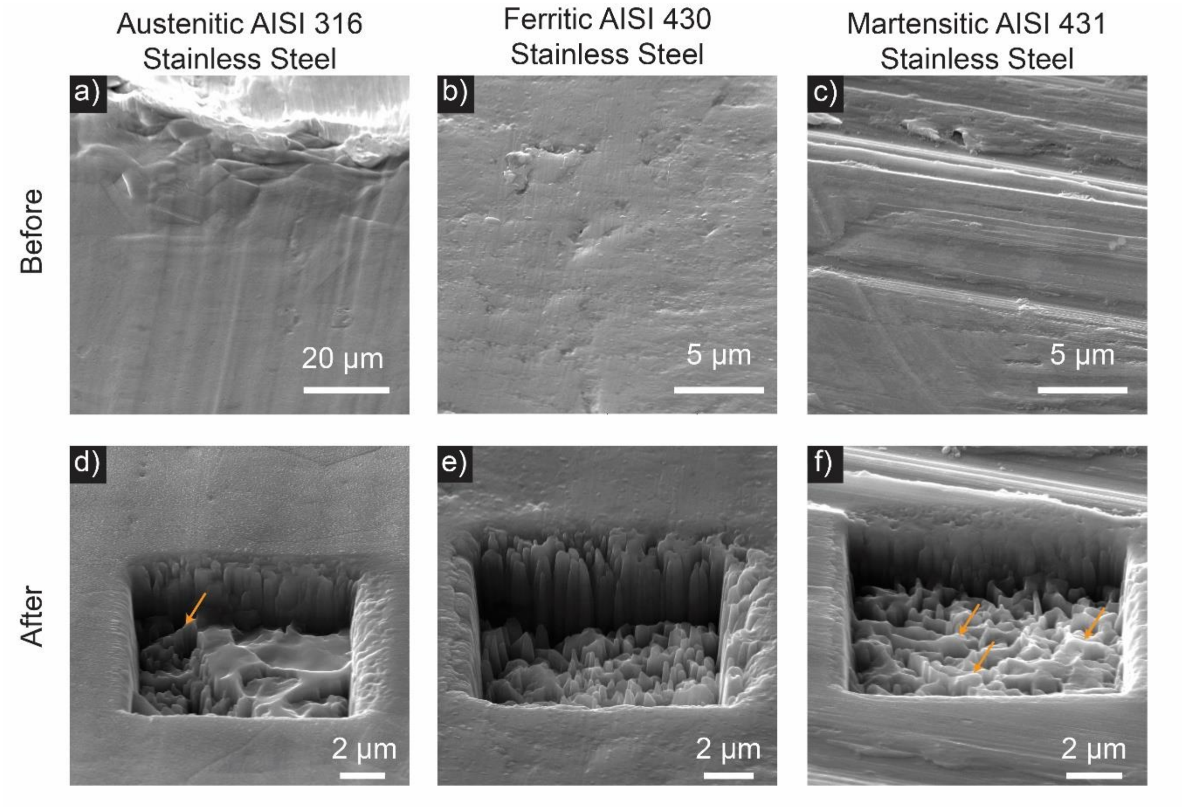

Figure S6. Gallium irradiation of austenitic AISI 316 (a, d), ferritic AISI 430 (b, e), and martensitic AISI 431 (c, f) stainless steel plates with a dose of 19.4 C/µm². a-c) Before and d-f) after gallium irradiation. Orange arrows denote the so-called nano-cliffs.
